# Presence of corrective saccades in patients with normal vestibulo-ocular reflex gain in video head impulse test

**DOI:** 10.3389/fneur.2023.1152052

**Published:** 2023-04-13

**Authors:** Kayoko Kabaya, Akina Fukushima, Sachiyo Katsumi, Toshiya Minakata, Shinichi Iwasaki

**Affiliations:** Department of Otolaryngology, Head and Neck Surgery, Nagoya City University Graduate School of Medical Science, Nagoya, Aichi, Japan

**Keywords:** video head impulse test, corrective saccade, caloric test, semicircular canal function, vestibulo-ocular reflex gain

## Abstract

**Background:**

The video head impulse test (vHIT) is a valuable clinical tool that can help identify dysfunction of the semicircular canals. While in cases with semicircular canal dysfunction, both decreased vestibulo-ocular reflex (VOR) gain and corrective saccades (CS) are usually observed, there are cases which show CS despite normal VOR gain in vHIT.

**Objective:**

This study aimed to investigate the clinical characteristics of patients who showed CS with normal VOR gain in vHIT.

**Materials and methods:**

Among 390 patients who underwent vHIT, 51 patients (20 males and 31 females, age 31–87 years, average 61.3 years old) who showed CS with normal VOR gain unilaterally during horizontal vHIT were included. All patients had normal vHIT (normal VOR gain and absent CS) on the contralateral side.

The VOR gain of vHIT, the maximum slow phase velocity in the caloric test, and the amplitude of cervical and ocular vestibular evoked myogenic potentials (cVEMPs and oVEMPs) were analyzed.

**Results:**

The VOR gain on the affected side (0.95 ± 0.08) was significantly smaller than that on the contralateral side (1.03 ± 0.13) in horizontal vHIT (*p* < 0.001). The maximum slow phase velocity in the caloric test on the affected side (17.9 ± 17.8 degrees/s) was significantly smaller than that on the contralateral side (21.3 ± 16.6 degrees/s, *p* = 0.020). There were no significant differences in the amplitude of cVEMPs or oVEMPs between the affected side and the contralateral side (*p* = 0.096 for cVEMP; *p* = 0.770 for oVEMP).

**Conclusion:**

The side that showed CS with normal VOR gain in horizontal vHIT showed significantly smaller VOR gain as well as smaller caloric responses compared to the contralateral side. Having CS with normal VOR gain could be a sensitive indicator of mild dysfunction of the semicircular canals.

## 1. Introduction

The video head impulse test (vHIT) is an objective method for testing vestibular function using brisk, passive rotations of the head in the plane of each semicircular canal (1). In healthy subjects, the vestibulo-ocular reflex (VOR) maintains gaze in space by compensation of the head rotation with an equal and opposite eye rotation. When subjects have vestibular dysfunction, the eye velocity is slower than the head-impulse velocity due to insufficient VOR, causing the eye to lag in the orbit, forcing the subject to make corrective saccades (CS) in order to re-fixate the target (1, 2). Thus, CS contribute to gaze stabilization by cooperating with residual VOR.

Two types of CS have been described. One is the “covert saccade” that occurs early during the rotation of the head and is almost imperceptible to the examiner. The other is the “overt saccade” that occurs after the head rotation has stopped and is easily observable by the clinician. While the bedside head impulse test allows the detection of overt CS only, vHIT as well as the search coil technique can detect and analyze both covert and overt CS (1, 3).

The main outcome parameters used in vHIT are VOR gain, the ratio of eye movement to head movement, and the presence of CS (1). In most cases with unilateral or bilateral dysfunction of the semicircular canals, both decreased VOR gain and CS are usually observed. However, in some cases, CS are observed in spite of normal VOR gain in vHIT, and the mechanism causing this is unknown. This study aimed to investigate the clinical characteristics of patients who showed CS with normal VOR gain in vHIT.

## 2. Materials and methods

### 2.1. Study design

This study was approved by the Research Ethics Committee, Graduate School of Medicine, Nagoya City University (#60-22-0800), and was conducted according to the tenets of the Declaration of Helsinki. Written informed consent was obtained from all participants.

### 2.2. Subjects

We retrospectively reviewed the medical records of 390 consecutive new patients who underwent vHIT at the Balance Disorder Clinic, Department of Otolaryngology and Head and Neck Surgery, Nagoya City University Hospital, from January 2020 to December 2021. Among them, 51 patients (13.1%, 20 males and 31 females, age range 31–87 years, mean age ± SD, 61.3 ± 14.3 years) who showed CS with normal VOR gain during the horizontal canal test in vHIT on one side and normal vHIT (normal VOR gain and absent CS) on the contralateral side were included. A detailed history was taken and a battery of tests was performed including a physical examination, neurological examination, pure-tone audiometry, and positional/positioning nystagmus testing under infrared CCD goggles. The vestibular function tests included vHIT, videonystagmography, caloric testing, and cervical and ocular vestibular evoked myogenic potentials (VEMPs). All of these vestibular function tests were performed on the same day. Neuroimaging studies such as computed tomography and magnetic resonance imaging of the brain were performed when considered necessary. The diagnostic criteria used in this study were: Meniere’s disease (4), vestibular neuritis (5), sudden deafness (6), benign paroxysmal positional vertigo (BPPV) (7), Ramsay Hunt syndrome (8), bilateral vestibulopathy (9), presbyvestibulopathy (3), and vestibular migraine (10).

### 2.3. Vestibular function tests

#### 2.3.1. Video head impulse test (vHIT)

The vHIT was performed to assess the vestibulo-ocular reflex (VOR) in the horizontal semicircular canal planes. Subjects were seated 1 m from a black fixation dot on a wall that served as the visual target. While the subject was asked to stare at the fixation dot, the examiner briefly and unpredictably rotated the subject’s head through a 10-to-20-degree angle. The head rotations were made in the horizontal plane with a peak head velocity between 100 and 250 degrees/s. The head impulses were repeated at least 15 times in each direction, and the eye and head velocities were recorded and analyzed using the Eye-See-Cam system (Interacoustics, Denmark). The VOR gains during vHIT were automatically measured using software that computed the slope of the regression between head and eye velocity (11). When a mean VOR gain in vHIT < 0.8, the horizontal semicircular canal was regarded as functionally abnormal (2, 11–13).

Corrective saccades (CS) were identified by their peak velocity. We only included CS that brought the eye toward the target with their peak velocity > 100 degrees/s in our analyses since tiny saccades were frequently observed in healthy subjects (2, 14). The amplitude of the saccade was measured at the peak of the velocity profile. Covert saccade velocity was adjusted such that the velocity of the VOR was removed (15, 16). We only included cases that showed CS in more than 50% of trials (17). Compensatory saccades that occurred during the head movement were classified as “covert.” Saccades appearing after the head movement had finished were classified as “overt.” The end of a head impulse was defined as the time when the head velocity reached zero for the first time.

#### 2.3.2. Caloric test

The caloric test was performed as a reference standard by irrigating the external auditory canal with 2 ml ice water (4°C) for 20 s followed by aspiration of water. This method of caloric stimulation is easier to perform than biothermal irrigation with water at 30°C and 44°C and has been shown to have a high sensitivity and specificity for detecting canal paresis (CP) based on Jongkee’s formula (18). Caloric nystagmus was recorded using electronystagmography. The CP% was calculated as the difference between the maximal slow phase velocity (MSPV) for each ear divided by the sum of slow-phase eye velocities. An abnormal caloric response was defined as having either of the following criteria: (i) CP% greater than 20% for unilateral dysfunction (19) or (ii) a MSPV < 10 degrees/s bilaterally for bilateral dysfunction (20).

#### 2.3.3. Cervical vestibular evoked myogenic potentials

Electromyographic (EMG) activity was recorded from a surface electrode placed on the upper half of each sternocleidomastoid muscle (SCM), with a reference electrode on the side of the upper sternum and a ground electrode on the chin. During the recording, in the supine position, subjects were instructed to raise their heads from the pillow to contract the SCM. The EMG signal from the stimulated side was amplified and bandpass-filtered (20–2,000 Hz) using Neuropack (Nihon Kohden, Tokyo, Japan). The stimulation rate was 5 Hz, and the analysis time was 100 ms. Short tone bursts of 500 Hz (95 dB normal hearing level, 135 dB SPL (peak value), rise/fall time 1 ms, plateau time 2 ms) were also presented. The latencies and amplitudes of the first positive–negative peaks (p13–n23) of the cVEMP were evaluated. Amplitude and latency were determined from the average of 2 runs. For the evaluation of amplitude, the asymmetry ratio for the p13–n23 amplitude (cVEMP AR) was calculated as 100*[(Au–Aa)/ (Aa + Au)], where Au is the p13–n23 amplitude on the unaffected side and Aa is the p13–n23 amplitude on the affected side (21). Based on the results from normal subjects, the upper limit of the cVEMP AR was set at 34.0% (21). Background EMG activity was monitored during recording, and subjects who had difficulty in maintaining SCM activity at a sufficient level (EMG activity > 150 μV) were treated as cases with no measurement results in the present study. When no reproducible p13–n23 was present in 2 runs, we regarded it as an “absent response.” When a reproducible p13–n23 was present and the cVEMP AR (%) was greater than the normal upper limit, we regarded it as a “decreased response.” An absent response or decreased response was treated as an abnormal response.

#### 2.3.4. Ocular vestibular evoked myogenic potentials

Subjects lay supine on a bed, with their head supported by a pillow and surface EMG electrodes placed on the skin 1 cm below (active) and 3 cm below (indifferent) the center of each lower eyelid. The ground electrode was placed on the chin. During testing, the subject looked up approximately 30 degrees above straight ahead and maintained their focus on a small dot approximately 1 m from their eyes. The signals were amplified by a differential amplifier (bandwidth: 0.5–500 Hz), and the unrectified signals were averaged (*n* = 50) using Neuropack (Nihon Kohden, Japan). The stimulation rate was 5 Hz, and the analysis time was 100 ms. Short tone bursts of 500 Hz (95 dB normal hearing level, 135 dB SPL (peak value), rise/fall time 1 ms, plateau time 2 ms) were also presented. We analyzed the first negative peak (nI) latency, the subsequent positive peak (pI) latency, and the amplitude between nI and pI. Amplitude and latency were determined from the average of 2 runs. For the evaluation of amplitude, the asymmetry ratio for nI–pI amplitude (oVEMP AR) was calculated as 100*[(Au–Aa)/(Aa + Au)], where Au is the nI–pI amplitude on the unaffected side and Aa is the nI–pI amplitude on the affected side (22). Responses recorded from the eye contralateral to stimulation were used for calculating oVEMP AR. Based on the results from normal subjects, the upper limit of normal oVEMP AR was set at 34.4 (22, 23). When no reproducible nI–pI was present in 2 runs, we regarded it as an “absent response.” When a reproducible nI–pI was present and the oVEMP AR (%) was greater than the normal upper limits, we regarded it as a “decreased response.” An absent response or decreased response was treated as an abnormal response.

### 2.4. Statistical analysis

The VOR gain of vHIT, the MSPV of the caloric test, and the amplitude of cVEMPs and oVEMPs were compared between the affected side with CS and the contralateral side using the Wilcoxon signed-rank test. Time from the onset of the disease, the VOR gain of vHIT, MSPV, and CP% of the caloric test were compared among the overt + covert groups, the isolated overt groups, and the isolated covert groups using the Kruskal–Wallis test. The occurrence of overt and covert saccades was compared using McNemar’s Chi-squared test. The abnormal rate of the caloric test was compared among the overt + covert groups, the isolated overt groups, and the isolated covert groups using Fisher’s exact test. Statistical analysis of the data was done using EZR (24). A difference was considered significant at *p* < 0.05.

## 3. Results

**Figure 1:**
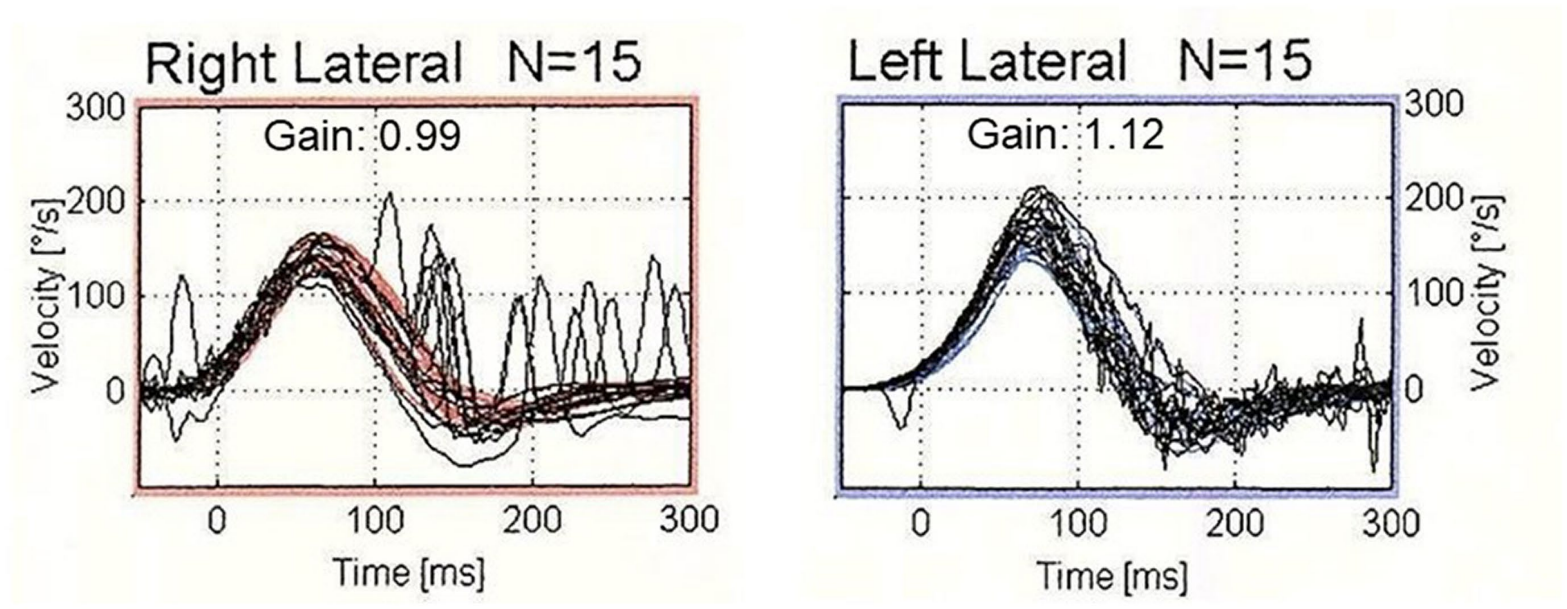
The results of video head impulse test (vHIT) in a typical patient who showed corrective saccades (CS) with normal vestibulo-ocular reflex (VOR) gain. vHIT showed normal VOR gain (0.99) with the presence of both covert and overt saccades during head impulses to the right horizontal semicircular canal. On the contralateral side, vHIT showed normal VOR gain (1.12) without CS.

Among 390 patients who underwent vHIT, 51 patients (20 males and 31 females: age range 31–87 years, mean age ± SD, 61.3 ± 14.3 years) showed CS with normal VOR gain unilaterally in horizontal vHIT and normal results on the contralateral side. Table 1 shows the clinical diagnoses of the 51 patients. The most common diagnosis was Meniere’s disease (*n* = 6; 11.8%), followed by vestibular neuritis (*n* = 5; 9.8%), sudden deafness with vertigo (*n* = 5; 9.8%) and benign paroxysmal positional vertigo (*n* = 4; 7.8%). Time from the onset of the disease was 420.4 ± 1082.9 days (range 4–7,305 days). Figure 1 shows the results of vHIT in a typical patient with Meniere’s disease who showed CS with normal VOR gain in horizontal vHIT.

**Table 1:** Clinical diagnosis of 51 patients who showed corrective saccades (CS) with normal vestibulo-ocular reflex (VOR) gain in video head impulse test (vHIT).

Diagnosis	Total *N* = 51	Overt + covert*N* = 17	Isolated overt *N* = 27	Isolated covert *N* = 7
Meniere’s disease	6 (11.8)	3 (17.6)	3 (11.1)	
Vestibular neuritis	5 (9.8)	2 (11.8)	3 (11.1)	
Sudden deafness with vertigo	5 (9.8)		5 (18.5)	
Benign paroxysmal positional vertigo	4 (7.8)		4 (14.8)	
Ramsay Hunt syndrome	2 (3.9)	1 (5.9)		1 (14.3)
Bilateral vestibulopathy	2 (3.9)	1 (5.9)	1 (3.7)	
Presbyvestibulopathy	2 (3.9)	1 (5.9)	1 (3.7)	
Vestibular migraine	2 (3.9)		1 (3.7)	1 (14.3)
Others	3 (5.9)	1 (5.9)	1 (3.7)	1 (14.3)
No established clinical entities	20 (39.2)	8 (47.1)	8 (29.6)	4 (57.1)

Data were shown as number (%), Overt + covert: patients with overt saccade and covert saccade, Isolated overt: patients with overt saccade only, Isolated covert: patients with covert saccade only.

Regarding the types of CS, 17 patients (33.3%) showed both overt and covert saccades (overt + covert), 27 (52.9%) showed isolated overt saccades, and 7 (13.7%) showed isolated covert saccades (Table 1). While Meniere’s disease and vestibular neuritis were most frequent in the “overt + covert” group, sudden deafness and benign paroxysmal positional vertigo were most frequent in the “isolated overt” group. On the other hand, patients having those peripheral vestibular diseases were rare in the “isolated covert” group. Time from the onset of the disease was 778.8 ± 1783.5 days in the “overt + covert” groups, 170.5 ± 329.6 days in the “isolated overt” groups, and 88.9 ± 129.1 days in the “isolated covert” groups. There were no significant differences in the time from the onset among those groups (*p* = 0.466).

**Figure 2:**
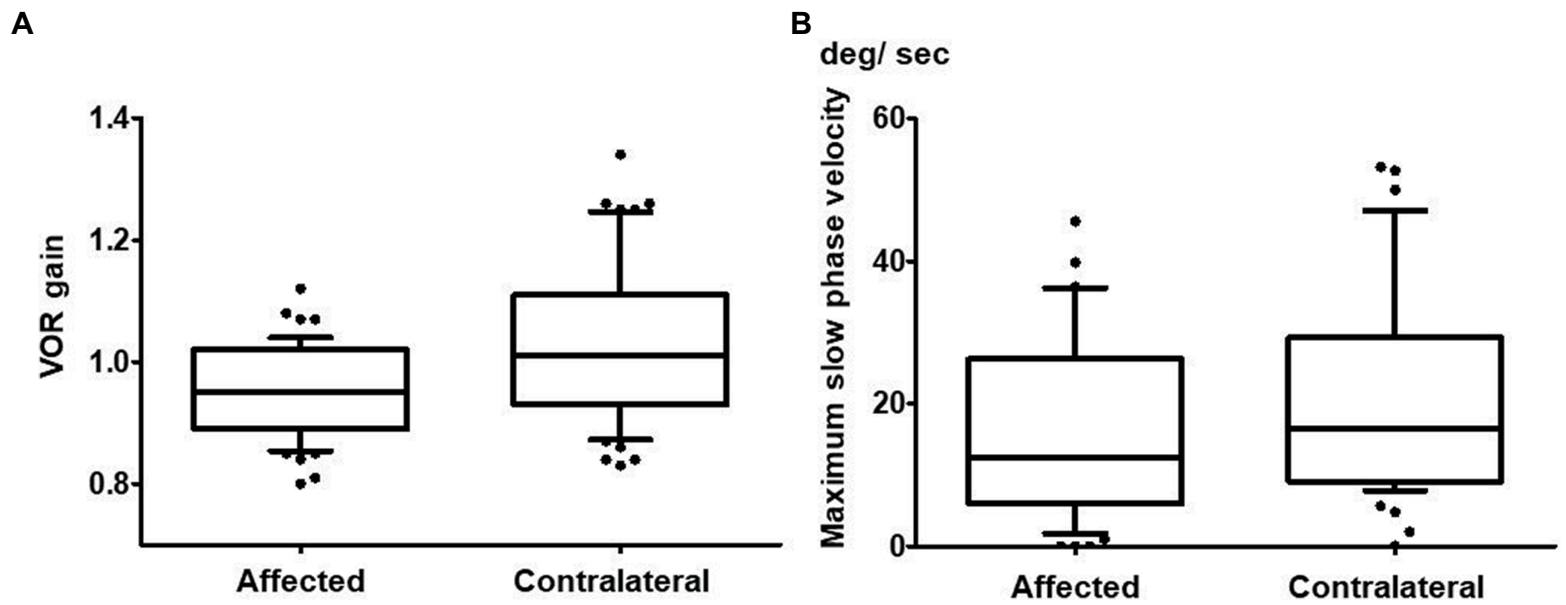
Vestibulo-ocular reflex (VOR) gain in vHIT and maximum slow phase velocity in the caloric test of the affected horizontal canal and the contralateral horizontal canal. **(A)** The mean VOR gain of the horizontal canal on the affected side was 0.95 ± 0.08 while it was 1.03 ± 0.13 for the horizontal canal on the contralateral side in vHIT (*n* = 51). This difference was significant (*p* < 0.001). **(B)** The maximum slow phase velocity on the affected side (17.9 ± 17.8 degrees/s) was significantly smaller than that on the contralateral side (21.3 ± 16.6 degrees/s) in the caloric test (*p* = 0.020; *n* = 50).

Tables 2, 3 show the results of the vestibular function tests including vHIT, caloric tests, cVEMPs, and oVEMPs in the 51 patients. The mean VOR gain of the horizontal vHIT was 0.95 ± 0.08 on the side with CS (affected side), while it was 1.03 ± 0.13 on the contralateral side (*n* = 51; Figure 2A). There was a significant difference between these values (*p* < 0.001). While both overt and covert saccades were observed, the occurrence of overt saccades was significantly greater than that of covert saccades (overt saccades: 86.3%, covert saccades: 47.1%; *p* < 0.001).

**Table 2:** Head impulse test (vHIT) and caloric test in patients who showed CS with normal VOR gain.

vHIT					
VOR gain	Type of CS	*N*	Affected side	Contralateral side	*p* Value
	All	51	0.95 ± 0.08	1.03 ± 0.13	< 0.001
	Overt + covert	17 (33.3%)	0.95 ± 0.07	1.05 ± 0.14	0.013
	Isolated overt	27 (52.9%)	0.96 ± 0.08	1.03 ± 0.13	0.005
	Isolated covert	7 (13.7%)	0.93 ± 0.07	0.98 ± 0.06	0.074
			*p* = 0.455^†^	*p* = 0.569^†^	
Caloric test					
MSPV (deg/s)	Type of CS	*N*	Affected side	Contralateral side	*p* Value
	All	50	17.9 ± 17.8	21.3 ± 16.6	0.020
	Overt + covert	16 (32.0%)	17.3 ± 12.5	19.7 ± 13.1	0.280
	Isolated overt	27 (54.0%)	20.2 ± 21.3	24.0 ± 18.6	0.099
	Isolated covert	7 (14.0%)	11.1 ± 13.9	14.9 ± 13.0	0.402
			p = 0.421^†^	*p* = 0.392^†^	
CP (%)	Type of CS	*N*	CP (%)	Normal/abnormal (unilateral/bilateral)	
	All	50	34.2 ± 27.9	27/23 (20/3)	
	Overt + covert	16 (32.0%)	37.4 ± 28.7	6/10 (10/0)	
	Isolated overt	27 (54.0%)	32.9 ± 26.4	11/16 (14/2)	
	Isolated covert	7 (14.0%)	31.9 ± 36.8	2/5 (4/1)	
			*p* = 0.701^†^	*p* = 0.922^†^	

† comparison of the overt + covert groups, the isolated overt groups, and the isolated covert groups.

Data are shown as *N* (%) or mean ± SD. vHIT: video head impulse test. VOR: vestibulo-ocular reflex. CS: corrective saccades. MSPV: maximum slow phase velocity. CP: canal paresis.

**Table 3:** Cervical vestibular evoked myogenic potentials (cVEMP) and ocular vestibular evoked myogenic protentials (oVEMP) in patients who showed CS with normal VOR gain.

Vestibular function tests		*p* Value
cVEMP (*N* = 49)
Amplitude (μV)		
Affected	137.4 ± 130.3	0.096
Contralateral	153.9 ± 148.6	
Normal	26 (53.1%)	
Abnormal	23 (46.9%)	
Unilateral	18 (36.7%)	
Bilateral	5 (10.2%)	
oVEMP (*N* = 39)
Amplitude (μV)		
Affected	5.2 ± 8.2	0.770
Contralateral	5.0 ± 7.3	
Normal	20 (51.3%)	
Abnormal	19 (48.7%)	
Unilateral	9 (23.1%)	
Bilateral	10 (25.6%)	

Data are shown as mean ± SD and/ or *N* (%). cVEMP; cervical vestibular evoked myogenic potential. oVEMP; ocular vestibular evoked myogenic potential.

The MSPV on the caloric test was 17.9 ± 17.8 degrees/s on the affected side, whereas it was 21.3 ± 16.6 degrees/s on the contralateral side. There was a significant difference between them (*p* = 0.020; *n* = 50; Figure 2B). Twenty patients (40.0%) showed abnormal caloric responses unilaterally, and 3 patients (6.0%) showed abnormal responses bilaterally. In all of the patients who showed unilaterally abnormal caloric responses, the side that showed abnormal caloric responses coincided with the affected side in horizontal vHIT. There were no significant differences in MSPV of either the affected side or the contralateral side among the three CS type groups (affected side: *p* = 0.421; contralateral side *p* = 0.392). The rate of abnormality on the caloric test was not different among those three groups (*p* = 0.922).

**Figure 3:**
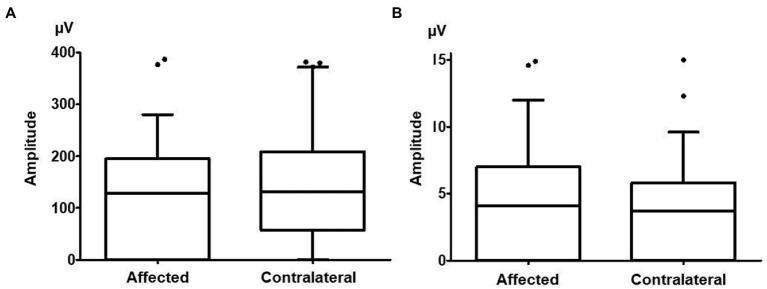
The cervical vestibular evoked myogenic potential (cVEMP) and ocular vestibular evoked myogenic potential (oVEMP) amplitudes on the affected side and contralateral side. **(A)** There were no significant differences between the amplitude of cVEMPs on the affected side (137.4 ± 130.3 μV) and that on the contralateral side (153.9 ± 148.6 μV, *p* = 0.096). **(B)** There were no significant differences between the amplitude of oVEMPs on the affected side (5.2 ± 8.2 μV) and that on the contralateral side (5.0 ± 7.3 μV, *p* = 0.770).

There were no significant differences in the amplitude of cVEMPs between the affected side (137.4 ± 130.3 μV) and the contralateral side (153.9 ± 148.6 μV, n = 49, *p* = 0.096; Figure 3A). Eighteen patients (36.7%) showed abnormal cVEMP responses unilaterally, and 5 patients (10.2%) showed abnormal responses bilaterally. In 12 of the 18 patients who showed abnormal cVEMPs unilaterally (66.7%), the side of the abnormal cVEMPs coincided with the affected side in vHIT.

There were no significant differences in the amplitude of oVEMPs between the affected side (5.2 ± 8.2 μV) and the contralateral side (5.0 ± 7.3 μV, *n* = 49, *p* = 0.770; Figure 3B). Nine patients (23.1%) showed abnormal oVEMP responses unilaterally, and 10 patients (25.6%) showed abnormal responses bilaterally. In 7 of the 9 patients who showed unilaterally abnormal oVEMPs (77.8%), the side of the abnormal oVEMPs coincided with the affected side in vHIT.

## 4. Discussion

In the present study, we retrospectively examined the characteristics of patients who showed CS with normal VOR gain unilaterally in horizontal vHIT. We showed that, in these patients, the VOR gain in vHIT as well as the caloric responses was significantly smaller on the affected side compared to those on the contralateral side, but that the amplitude of the oVEMP or cVEMP was not different between the sides. These results suggest that slight semicircular canal hypofunction, even within the normal range of VOR gain, can induce CS in vHIT.

Several previous studies showed that CS occur in subjects with normal VOR gain (2, 25, 26). Yang et al. (2) reported that CS occurred in approximately 50% of normal subjects with no significant difference in the rate among age groups. Rambold et al. (26) reported that CS occurred in approximately 30% of normal subjects, and the frequency of CS increased significantly with increasing age. These studies included CS with very small velocity (e.g., less than 5 degrees/s) as well as CS that took the eye in the opposite direction to the target (2, 15, 26). In the present study, we included only CS that brought the eye toward the target with a peak velocity of > 100 degrees/s since tiny CS or CS in the same direction as the head movement are not pathological (14, 26).

The most common clinical diagnosis in the patients who showed CS with normal VOR gain in horizontal vHIT in the present study was Meniere’s disease, followed by vestibular neuritis. This result is consistent with a previous study in which patients who showed CS with normal VOR gain in horizontal vHIT were retrospectively analyzed (27). While it has been reported that patients with Meniere’s disease tend to show normal VOR gain in vHIT but reduced caloric responses (28), it is unknown whether an increased diameter of the semicircular canal due to endolymphatic hydrops affects the generation of CS in vHIT. Regarding vestibular neuritis, it has been shown that a portion of patients show CS even after full recovery of the VOR during the follow-up (29, 30). All the patients with vestibular neuritis included in the present study underwent vestibular function testing more than 6 months after the onset of the disease.

In the present study, we defined a mean VOR gain of < 0.8 as abnormal in horizontal vHIT based on previous studies that measured VOR gain in vHIT in healthy subjects (2, 11–13). It is possible that VOR gain in vHIT is affected by the patient’s age and the peak head velocity. McGarvie et al. recorded vHIT in a large sample of healthy subjects and showed that age is not a statistically significant factor in VOR gain in healthy subjects even in to their 80 s except for a small decrease in VOR gain for the posterior canal (13). They also showed that VOR gain evoked by a wide range of peak head velocities (100–250 degrees/s) is not affected by the subject’s age. We included horizontal vHIT recordings evoked by impulses with a peak head velocity between 100 and 250 degrees/s in our analysis.

In the present study, overt saccades were more frequently observed than covert saccades in patients with normal VOR gain in horizontal vHIT. Since retinal slip during high-velocity head impulses has been suggested as the main trigger for overt saccades (31, 32), it is possible that the tiny retinal slip caused by a slight reduction of VOR gain during head impulses might trigger the generation of overt saccades. On the other hand, covert saccades have been suggested to play an important role in facilitating recovery in visual performance during head movements (33). Saccadic suppression, in which visual processing is suppressed so as not to perceive the motion of the eye during saccades, contributes to reduce oscillopsia and improve visual performance during high-velocity head movements (33, 34). Previous studies have shown that patients suffering from acute unilateral vestibular loss show both overt and covert saccades at the acute stage, but the proportion of covert saccades increases through the recovery period (15, 30, 35). The mechanism of the generation of covert saccades is still unclear because their latencies are too short to be generated by visual input. The remaining vestibular function, the somatosensory input from the neck, and the internal models in the central nervous system have all been proposed as possible triggers for covert saccades (36–38). It is possible that the mechanism of generation of covert and overt saccades is different among the patients in this study due to factors such as variations in the patients’ age, the cause of vestibular hypofunction and its duration, and the extent and the degree of the remaining vestibular function. To clarify these factors, analysis of a larger number of patients with CS and normal VOR gain is needed.

There were no significant relationships between the presence of CS in vHIT and the results of oVEMPs or cVEMPs in the present study. It has been shown that cVEMPs reflect the function of the saccule and the inferior vestibular nerve systems, whereas oVEMPs reflect the function of the utricle and the superior vestibular nerve systems (39). Since both horizontal vHIT and oVEMPs reflect the function processed by the superior vestibular nerve, the absence of the association between the results of the vHIT and the oVEMP suggests that the lesions generating CS in vHIT in the patient group included in the present study might lie in the semicircularcanals.

There are several limitations in the present study. First, the number of patients was insufficient, and the patients’ age, clinical diagnoses, and duration of the disease varied widely. A future study with large sample sizes will be needed to further confirm our results. Second, we only analyzed the horizontal canals, not the vertical semicircular canals, because the results of horizontal vHIT were more reliable than those of the vertical canals. The head rotation technique was more stable and artifacts were less frequent in the plane of the horizontal canal in comparison with that of the vertical canals. In addition, the data from the caloric test are available for evaluation of the function of the horizontal semicircular canal. Since the appearance rate of CS is different between each semicircular canal (40), different results will be obtained by analysis of the vertical canals. Last, we only recruited patients who showed CS with normal VOR gain unilaterally. It is possible that patients who show CS with normal VOR gain bilaterally have different mechanisms of generating CS. While we showed mild dysfunction of the semicircular canals can generate CS, it is possible that other mechanisms such as the central vestibular dysfunction may have an association with the generation of CS. Future studies are needed to investigate the clinical characteristics of such patients.

## 5. Conclusion

We investigated the clinical characteristics of patients who showed CS with normal VOR gain unilaterally in horizontal vHIT. We revealed that the side showing CS had a significantly smaller VOR gain as well as smaller caloric responses as compared to the contralateral side, suggesting that a slight reduction in semicircular canal function can generate CS in vHIT. We postulate that having CS with normal VOR gain in vHIT could be a sensitive indicator of mild dysfunction of the semicircular canals.

## Data availability statement

The raw data supporting the conclusions of this article will be made available by the authors, without undue reservation.

## Ethics statement

The studies involving human participants were reviewed and approved by the Research Ethics Committee, Graduate School of Medicine, Nagoya City University (#60-22-0800). The patients/participants provided their written informed consent to participate in this study.

## Author contributions

KK, AF, SK, TM, and SI conceived and designed the study, wrote, and revised the manuscript. KK, AF, and SI collected data and analyzed. All authors contributed to the article and approved the submitted version.

## Funding

This work was supported by the Ministry of Education, Culture, Sports, Science and Technology (20K11161, 21H03088).

## Conflict of interest

The authors declare that the research was conducted in the absence of any commercial or financial relationships that could be construed as a potential conflict of interest.

## Publisher’s note

All claims expressed in this article are solely those of the authors and do not necessarily represent those of their affiliated organizations, or those of the publisher, the editors and the reviewers. Any product that may be evaluated in this article, or claim that may be made by its manufacturer, is not guaranteed or endorsed by the publisher.
